# Different population trajectories of two reef‐building corals with similar life‐history traits

**DOI:** 10.1111/1365-2656.13463

**Published:** 2021-03-17

**Authors:** Tom Shlesinger, Robert van Woesik

**Affiliations:** ^1^ Institute for Global Ecology Florida Institute of Technology Melbourne FL USA

**Keywords:** coral abundance, coral reefs, demography, integral projection model, life‐history traits, population dynamics, population trajectories, Red Sea

## Abstract

1. Increases in the frequency and intensity of acute and chronic disturbances are causing declines of coral reefs world‐wide. Although quantifying the responses of corals to acute disturbances is well documented, detecting subtle responses of coral populations to chronic disturbances is less common, but can also result in altered population and community structures.
2. We investigated the population dynamics of two key reef‐building Merulinid coral species, *Dipsastraea favus* and *Platygyra lamellina*, with similar life‐history traits, in the Gulf of Eilat and Aqaba, Red Sea from 2015 to 2018, to assess potential differences in their population trajectories.
3. Demographic processes, which included rates of survival, growth, reproduction and recruitment were used to parametrize integral projection models and estimate population growth rates and the likely population trajectories of both coral species.
4. The survival and reproduction rates of both *D. favus* and *P. lamellina* were positively related to coral colony size, and elasticity analyses showed that large colonies most influenced population dynamics. Although both species have similar life‐history traits and growth morphologies and are generally regarded as ‘stress‐tolerant’, the populations showed contrasting trajectories—*D. favus* appears to be increasing whereas *P. lamellina* appears to be decreasing.
5. As many corals have long‐life expectancies, the process of local and regional decline might be subtle and slow. Ecological assessments based on total living coral coverage, morphological groups or functional traits might overlook subtle, species‐specific trends. However, demographic approaches capable of detecting subtle species‐specific population changes can augment ecological studies and provide valuable early warning signs of decline before major coral loss becomes evident.

## Graphical abstract

This study investigated coral demographic processes to parametrize integral projection models and predict future trends of coral populations. It demonstrates that coral species possessing similar life‐history traits and growth morphologies may have contrasting trajectories. Such approaches can provide early warning signs of population decline before major coral loss becomes evident.

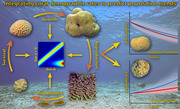

## 1 INTRODUCTION

Supporting over 800 reef‐building coral species, and hundreds of thousands of associated organisms, coral reefs are the most diverse marine ecosystem (Cairns, 1999; Knowlton et al., 2010). Coral reefs also provide valuable goods and services to human communities (Hoegh‐Guldberg et al., 2007; Hughes et al., 2017; Knowlton, 2001). However, coral reefs are undergoing dramatic declines (Hoegh‐Guldberg et al., 2007; Hughes et al., 2017; Pandolfi et al., 2003) because of acute and chronic disturbances. For example, the increasing frequency and intensity of thermal‐stress events are acute disturbances leading to coral bleaching, disease and mortality (Ainsworth et al., 2016; Gilmour et al., 2013; Graham et al., 2015; Loya et al., 2001; Riegl et al., 2018; Stuart‐Smith et al., 2018; Sully et al., 2019). In addition, land‐use changes, pollution, reduced water quality and other local impacts are examples of chronic disturbances contributing to coral population declines (Abelson, 2020; Kennedy et al., 2013; MacNeil et al., 2019; Pandolfi et al., 2003). While changes on coral reefs can take many forms, most studies report coral responses to acute disturbances and provide useful information on both local and regional differences in coral decline (Frade et al., 2018; Hoogenboom et al., 2017; Mies et al., 2020; van Woesik, Houk, et al., 2012). Yet, subtle, chronic disturbances to coral‐reef systems might frequently go unnoticed (Hartmann et al., 2018; Mumby, 2017; Shlesinger & Loya, 2019), although they too can lead to community homogenization, declines in species diversity, impaired recovery, and changes in population abundance and structure (Bak & Meesters, 1999; Ortiz et al., 2018; Osborne et al., 2017; Riegl et al., 2012).

Subtle, slow changes in coral populations are often difficult to detect because they require detailed demographic studies on population‐ and individual‐level vital rates, such as survival, growth, reproduction, and recruitment. Yet, if subtle changes to populations are detected early enough, they may trigger mitigating policies that could improve the conditions from which the at‐risk corals may recover. Here we examine the population demography of two massive Merulinid coral species, *Dipsastraea favus* and *Platygyra lamellina,* to estimate the sensitivities of the populations to a suite of demographic processes and to predict the likely trajectories of these key species on the reefs of the Gulf of Eilat and Aqaba, Red Sea.

Despite the value of coral cover as a key metric of assessing a coral‐reef state, changes in coral cover alone cannot accurately predict species‐specific population trajectories (Edmunds & Riegl, 2020; Hartmann et al., 2018; Ortiz et al., 2018; Pisapia et al., 2020; Shlesinger & Loya, 2019). For example, coral composition at any one location can change from one assemblage to another, or change from high to low diversity, or from a few large colonies to many small colonies—while not displaying obvious changes in coral cover (Done, 1999; Edmunds & Riegl, 2020; Fine et al., 2019; González‐Barrios et al., 2020; Knowlton, 2001; Pisapia et al., 2020). Yet those alternate communities are fundamentally different from each other. Additionally, some coral species exhibit high survival rates, slow growth rates and long‐life expectancies although their recovery from disturbances may take decades. By contrast, other coral species exhibit low survival rates, fast growth rates and short‐life expectancies but may recover rapidly from disturbances. Therefore, information on key demographic processes is needed to accurately predict the fate of coral populations, especially in modern times when coral abundances are globally declining.

Traditional life tables and matrix projection models are widely used in population biology to quantify change (Caswell, 2001; Lefkovitch, 1965; Leslie, 1945). Such tables and models continue to provide valuable insights into the dynamics of coral populations (Doropoulos et al., 2015; Pisapia et al., 2020; Riegl et al., 2018). These models, however, use discrete life stage or size classes and have been recently improved by the development of integral projections models (IPMs) that allow more flexibility in model construction and use continuous sizes—thus avoiding sensitivities stemming from the choice of discrete life stages or size classes (Coulson, 2012; Easterling et al., 2000; Edmunds et al., 2014; Ellner & Rees, 2006; Merow et al., 2014; Rees et al., 2014). Given their strengths, IPMs are being increasingly implemented in studies of coral populations and have been instrumental in: (a) studying coral population responses to the impacts of disease (Bruno et al., 2011), (b) detecting recovery from disturbances (Kayal et al., 2018), (c) determining responses to restoration (Montero‐Serra et al., 2018), (d) examining population responses to environmental changes (Cant et al., 2020; Edmunds et al., 2014; Elahi et al., 2016; Madin et al., 2012) and (e) assessing the viability and dynamics of populations (Precoda et al., 2018; Scavo Lord et al., 2020). The IPM framework allows for a simple and flexible incorporation of demographic processes relative to the size of coral colonies. These demographic processes may include the probability of survival, the rate of colony growth, colony fecundity and the number of recruits, which are integrated across different monitoring intervals to approximate the likely rates of population growth. Such demographic processes can be further evaluated to determine their influence on rates of coral population growth.

Here, we use an IPM framework to examine the demographic processes that influence two key reef‐building coral species, *Dipsastraea favus* and *Platygyra lamellina* on the reefs of Eilat, Red Sea from 2015 to 2018. Specifically, the objectives of this study were to: (a) use a suite of demographic processes to determine the expected rates of population growth (λ) of the corals, (b) determine the sensitivities of the population growth rates of the corals to different demographic processes, (c) compare population growth rates and their sensitivities between two massive, slow growing coral species that share similar life‐history traits and growth morphology, and (d) predict the likely population trajectories of the corals.

## 2 MATERIALS AND METHODS

### 2.1 Demographic monitoring and estimates of vital rates

We monitored colonies of two common Indo‐Pacific coral species, *Dipsastraea favus* (*n* = 167) and *Platygyra lamellina* (*n* = 83) in the Gulf of Eilat and Aqaba, Red Sea from 2015 to 2018. These two coral species are both massive, slow growing, hermaphroditic, broadcast‐spawning species belonging to the family Merulinidae. In 2015, 10 × 3 m^2^ permanently marked reef plots were haphazardly positioned at 4–5 m depth. The plots were photographed and all corals within the plots were identified and mapped using Adobe Photoshop CS6. The coral colonies were followed through to 2018 to estimate survival, growth and recruitment. To estimate size‐specific survival and growth, we measured the diameter (cm) of all colonies within the plots at the beginning of the study in 2015 and at the end of the study in 2018. Since both coral species tend to grow as symmetrical, dome‐shaped massive colonies, we measured the diameter of each colony as a proxy of coral colony size. We used a census interval of 3 years because of the slow growth rates of the two massive coral species. To estimate recruitment, all new corals, which were present in the plots in 2018, but absent from the 2015 maps, were considered to be recruits. As some recruits were too small to be identifiable to species level in the field, the recruitment rates were most likely underestimates.

To estimate reproductive traits as a function of colony size for the two coral species we used data measured by Shlesinger (1985) and gathered by colony size classes (Table S1). These data included proportions of reproductive colonies, proportions of reproductive polyps within colonies and maximum fecundity per polyp. For the analysis, and based on the data collected, we used random draws from a Gaussian distribution based on the group's mean size and standard deviation. Similarly, we used random draws from a Bernoulli distribution to determine whether a colony was reproductive or not and used random draws from a Gaussian distribution to determine the proportion of reproductive polyps. Colony fecundity was estimated as the product of colony surface area (cm^2^), number of polyps per cm^2^ and polyp fecundity. The number of polyps per cm^2^ was calculated by counting the number of polyps in small quadrats (9 cm^2^ in size) on 10 colonies of each species. Colonies of both species were considered as symmetrical hemispheres and their surface areas (*S*) were calculated as

(1) $$S=2\pi r^{2},$$

where *r* is the radius (cm) of the colony. By using reproductive data from a former study, we made an assumption that the fecundity of the studied coral species has not changed through time. This might be a reasonable assumption, and the approach was adopted in previous demographic studies (Cant et al., 2020; Elahi et al., 2016; Precoda et al., 2018). However, fecundity may have changed through time. Previous studies assessing sublethal effects of chronic disturbances on coral reproduction showed reductions in the percentage of reproducing colonies, reductions in the proportion of gravid polyps within colonies and reductions in polyp fecundity, leading to two‐ to fivefold reductions in reproductive output under stressful conditions (Hartmann et al., 2018; Loya et al., 2004; Tomascik & Sander, 1987). Therefore, to account for a potential decline in fecundity through time, we constructed additional IPMs with a twofold reduction in fecundity.

Survival was modelled using a logistic regression (i.e. generalized linear models with a binomial link function) as a function of colony size in 2015. Growth was modelled using a linear regression examining the relationship between colony size in 2015 and 2018. The probability of a colony being reproductive and the proportions of reproductive polyps within colonies were modelled using logistic regressions against colony size. The estimated numbers of oocytes per colony were based on count data and were modelled using a Poisson regression. However, since our initial Poisson models were overdispersed, we used a negative binomial model for the number of oocytes per colony relative to colony size, which improved the models’ diagnostics. We used natural log‐transformed size data for all models.

### 2.2 Constructing integral population models

We constructed integral projection models (IPMs) incorporating demographic functions that described size‐dependent coral survival, growth, reproduction and recruitment, to estimate the population asymptotic growth rate (λ). The general mathematical form of the IPM is:

(2) $$n\left(z^{\prime },t+3\right)=\int_{\mathrm{Lower}}^{\mathrm{Upper}}K\left(z^{\prime },z\right)n\left(z,t\right)dz,$$

where *z*′ indicates the colony size at time *t* + 3 (i.e. in 2018) and *z* is the colony size at time *t* (i.e. in 2015). The size distribution *n*(*z*′, *t* + 3) is estimated as a function of the colony size distribution *n*(*z*, *t*) of individuals of all colony sizes *z* at time *t* integrated across the range of colony sizes from *Lower* to *Upper* bounded colony sizes. The IPM kernel *K*(*z*′, *z*) relates the colony size distribution at time *t* to a colony size distribution at time *t* + 3 using different functions describing how individual colonies survive, grow, reproduce and recruit. The kernel (*K*) can be split into two sub‐kernels (Equation 3). The first sub‐kernel (i.e. for survival, *s*, and growth, *g*) describes the probability distribution that an individual colony size can become at *t + *3, conditioned on whether it survived the census interval. The second sub‐kernel (i.e. for reproduction and recruitment, *r*) describes the number of potential offspring produced during the census interval and their colony size distribution, as:

(3) $$K\left(z^{\prime },z\right)=s\left(z\right)g\left(z^{\prime },z\right)+r\left(z^{\prime },z\right).$$

The reproduction and recruitment sub‐kernel *r*(*z*′, *z*) describes the production of new recruits and their size distributions by reproductive colonies, and was the product of:

(4) $$r\left(z^{\prime },z\right)=P_{\text{colony}}\left(z\right)P_{\text{polyps}}\left(z\right)f_{\text{oocytes}}\left(z\right)f_{\text{recruits}}\left(z^{\prime }\right)P_{\text{establishment}\_\text{ratio}},$$

where *P*_colony_(*z*) is the probability that a colony is reproductive as a function of colony size *z*, *P*_polyps_(*z*) is the proportion of reproductive polyps within a colony as a function of colony size *z*, *f*_oocytes_(*z*) is the potential maximum number of oocytes produced as a function of colony size *z*, *f*_recruits_(*z*′) is the size distribution of recruits observed at *t* + 3 and *P*_establishment_ratio_ is the ratio of recruits observed at *t + *3 compared with the potential oocyte production at time *t*.

We used the midpoint rule as the integration method (Ellner & Rees, 2006) to evaluate the IPM kernels along a grid of 300 mesh points (changing the number of mesh points from 50 to 500 did not affect the shape of the IPMs and the estimation of λ, indicating that the number of mesh points was adequate). The IPMs were discretized into their lower and upper limits within the colony‐size range by calculating 0.9 times the diameter (cm) of the smallest observed coral colony of each species as the lower limit, and then 3.912 or 4.605 (on a natural log scale), which equates to a colony diameter of 50 cm for *D. favus* and 100 cm for *P. lamellina*, respectively, as the upper limits. These colony‐size ranges span over all coral colony sizes observed in this study, and across larger colony sizes than observed to avoid colonies being evicted from the model (Merow et al., 2014). As we did not observe mortality among the largest colonies in this study (only one colony with a diameter larger than 10 cm, which is 2.3 on natural log scale, went through whole‐colony mortality), the logistic regressions of survival can reach the asymptote of 1, effectively predicting immortality (Merow et al., 2014). Therefore, to limit this implausible biological condition, we introduced a size‐independent mortality probability of 1% following Edmunds et al. (2014) and Precoda et al. (2018).

The intrinsic rates of population growth (λ) were estimated by calculating the dominant eigenvalues of the IPMs, where λ < 1 indicates population decline and λ > 1 indicates population growth. Elasticity and sensitivity analyses were conducted to determine the contribution of the different demographic processes and colony‐size transitions on the population growth rates (λ). To further examine how each demographic process influenced the population growth rates (λ) we conducted perturbation analyses (Coulson et al., 2010) by independently increasing and decreasing each demographic process coefficient by 1% and constructing new IPMs to calculate the proportional change in the population growth rates (λ). All analyses were performed using r v4.0.2 (R Core Team, 2020), and all code and data can be accessed at: http://github.com/TomShlesinger/Different_coral_population_trajectories (Shlesinger, 2021).

### 2.3 Projecting population trajectories

To predict the likely long‐term population dynamics, we used stochastic projections based on each coral population's growth rate value λ derived from the IPMs. We computed 500 projections for each coral species by randomly assigning λ values drawn from a Gaussian distribution using the IPM’s λ value as the mean and using an estimate of variance by simultaneously perturbing all demographic process coefficients by ±1%. To simulate the effect of reduced fecundity on population dynamics we constructed new IPMs, using a regression of colony fecundity as described above while reducing the number of oocytes per colony to half of the original values. We then computed an additional set of 500 projections for each coral species based on the new λ values and compared the two sets of projections (i.e. projections based on the original fecundity values versus fecundity reduced to half).

## 3 RESULTS

**FIGURE 1:**
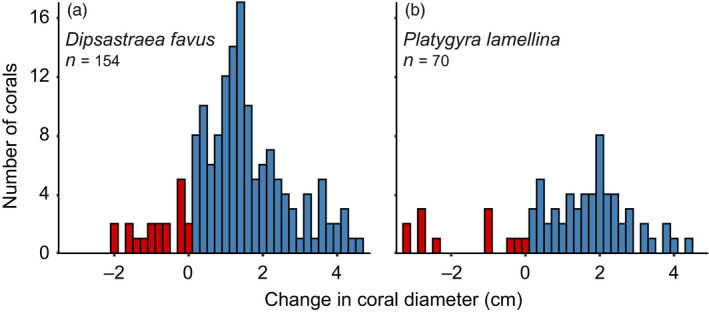
Change in coral diameters from 2015 to 2018, indicating growth (depicted in blue) and shrinkage (depicted in red) of two massive corals (a) *Dipsastraea favus* and (b) *Platygyra lamellina* on the reefs of the Gulf of Eilat and Aqaba, Red Sea

**FIGURE 2:**
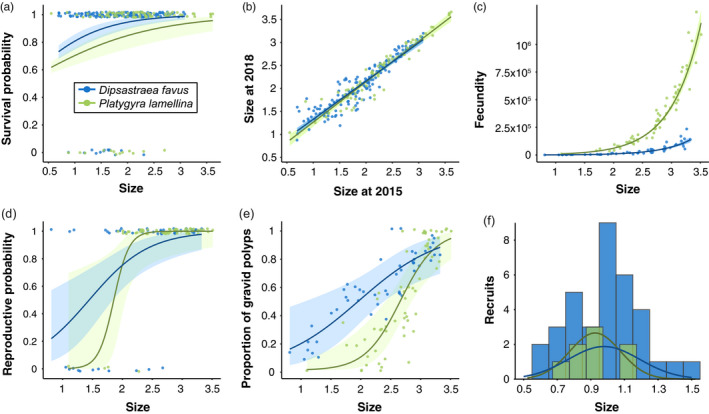
Demographic rates as a function of coral size for both *Dipsastraea favus* (depicted in blue) and *Platygyra lamellina* (depicted in green) on the reefs of the Gulf of Eilat and Aqaba, Red Sea from 2015 to 2018. Points represent individual colony data and the curves represent the fitted functions with 95% confidence intervals as the shaded areas. Size is given on log scale. (a) Probability of survival using a logistic regression. (b) Growth using a linear regression. (c) Fecundity (i.e. number of oocytes) per colony using a negative binomial regression. (d) Probability of a colony being reproductive using a logistic regression. (e) Proportion of gravid polyps within a colony using a logistic regression. (f) Size frequency distribution of the observed recruits and the normal distribution fitted to the data. Note that the histogram of *P. lamellina* recruits is presented with a small horizontal offset to aid visualization

During the 3‐year study period (from 2015 to 2018), more than 80% of the coral colonies that survived experienced net growth, whereas less than 20% of the coral colonies decreased in size or went through partial mortality (Figure 1). The average growth rates were similar for both species (i.e. 1.67 ± 1.08 and 1.82 ± 1.01 cm, for *Dipsastraea favus* and *Platygyra lamellina* respectively). Survivorship and reproductive traits showed positive relationships with colony size for both species (Figure 2; Tables S2 and S3). Small colonies (equating to ≤10 cm in diameter) had a lower probability of survival than large colonies (equating to >10 cm), such that almost all the mortalities of both species were evident for colonies ≤10 cm in diameter (which is 2.3 on the natural log scale in Figure 2a). Colony fecundity increased exponentially with colony size (Figure 2c). The probability of being reproductive and the proportion of reproductive polyps also increased with colony size (Figure 2d,e). There were considerably fewer *P. lamellina* recruits in the monitored reef plots than there were *D. favus* recruits (Figure 2f).

**FIGURE 3:**
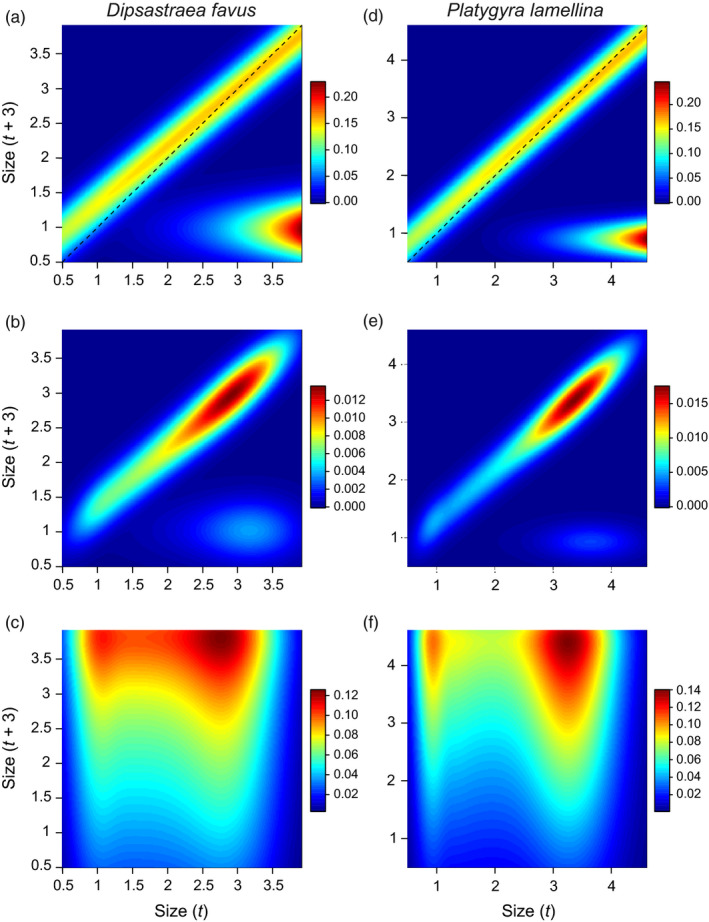
Integral projection model output for the massive corals (a–c) *Dipsastraea favus* and (d–f) *Platygyra lamellina* on the reefs of the Gulf of Eilat and Aqaba, Red Sea from 2015 to 2018. (a) and (d) depict the whole integral projection model kernel with warmer colours indicating greater probability of size transitions from one year to the next. The dashed lines represent the 1:1 slope (i.e. no change in size between years). (b) and (e) depict the integral projection models’ elasticities, and (c) and (f) are the models’ sensitivities. The diagonal high probability ridges in (a,b) and (d,e) reflect the survival and growth sub‐kernel, and the high probability ‘hotspots’ on the bottom right in (a,d) represent the reproduction and recruitment sub‐kernel

The highest probabilities of survival and growth were evident for large colonies, which also contributed the most to recruitment (via reproduction) (Figure 3a,d). The IPM of *D. favus* yielded a positive population growth rate, with λ = 1.071, which suggests that the population grew by 7.1% within the study's 3‐year study period. By contrast, the IPM of *P. lamellina* had a negative population growth rate, with λ = 0.957, which suggests that the population declined by 4.3% during the same time period.

**FIGURE 4:**
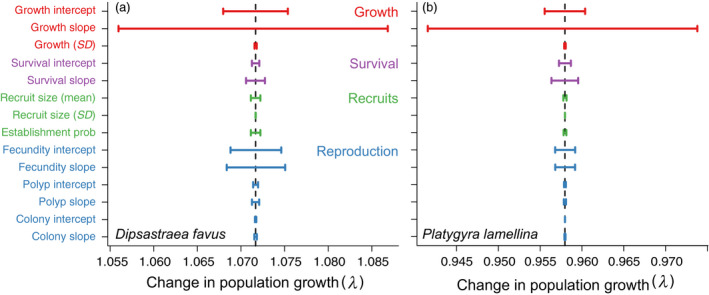
Perturbation analysis of the massive corals (a) *Dipsastraea favus* and (b) *Platygyra lamellina* integral projection models (IPMs) on the reefs of the Gulf of Eilat and Aqaba, Red Sea from 2015 to 2018. The analysis is a consequence of a ± 1% perturbation to each of the parameters (i.e. the regression coefficients) used to construct the IPMs for the population growth rate (λ), where *SD* = standard deviation

Elasticity analyses showed that large colonies contributed the most to the population growth rates, mostly through survival and growth (Figure 3b,e). Estimating the IPM sub‐kernels elasticities separately (Figure S1) revealed that reproduction/recruitment contributed ~5% to the population growth rate of *D. favus*, whereas reproduction/recruitment contributed only ~2% to the population growth rate of *P. lamellina*. Sensitivity analysis again suggested that population growth rate was most sensitive to changes in the largest colonies of both species, and to a lesser extent the smallest colonies of both species (Figure 3c,f). The perturbation analyses revealed that colony growth had the largest impact on population growth rates, followed by fecundity, although fecundity had a larger impact on *D. favus* population growth rate than on *P. lamellina* (Figure 4).

**FIGURE 5:**
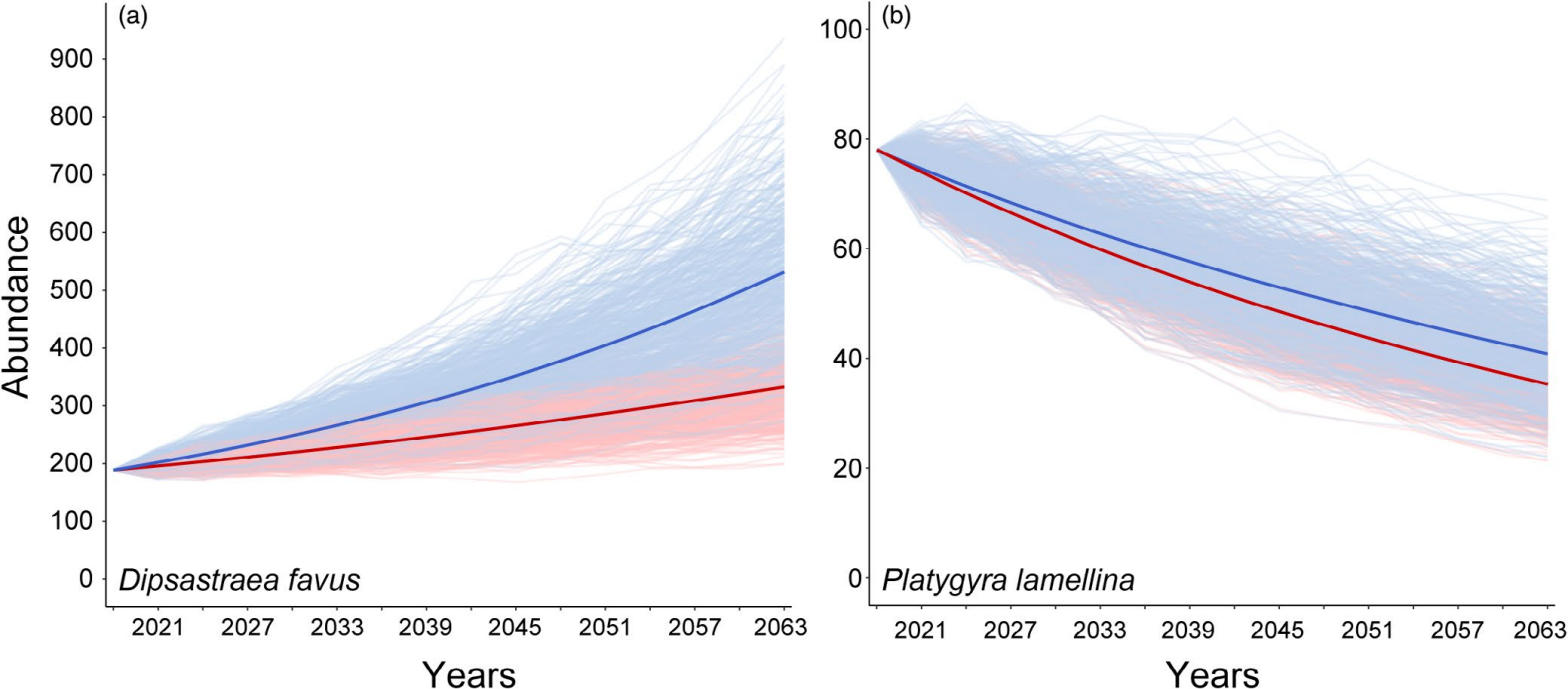
Predicted population dynamics of the massive corals (a) *Dipsastraea favus* and (b) *Platygyra lamellina* on the reefs of the Gulf of Eilat and Aqaba, Red Sea from 2018 to 2063. Blue lines represent the mean of 500 stochastic projections (depicted as thin light blue lines) based on the population growth rate (λ) derived from the integral projection models (IPMs) for the original fecundity values (λ = 1.071 for *D. favus* and λ = 0.957 for *P. lamellina*). Red lines represent the mean of 500 stochastic projections (depicted as thin light red lines) based on the population growth rate (λ) derived from the IPMs with fecundity reduced to half (λ = 1.038 for *D. favus* and λ = 0.948 for *P. lamellina*)

The projections of the population dynamics of *D. favus* suggest that the population will most likely increase and might even double within ~30 years (Figure 5a). By contrast, the projections of the population dynamics of *P. lamellina* suggest that the population will slowly decrease and might halve within ~40 years (Figure 5b). The projections of both species were sensitive to the simulation of reduced fecundity and differed from the projections using the original fecundity values (generalized linear mixed models, *p* < 0.001 for both). However, the difference between the projections based on original fecundities versus fecundities reduced by 50% were much more subtle for *P. lamellina* than for *D. favus* (Figure 5).

## 4 DISCUSSION

This 3‐year study in the Gulf of Eilat and Aqaba, Red Sea shows that the populations of two coral species, which possess similar traits and growth morphologies, have contrasting population trajectories despite an overall increase in total coral cover at the same location (Shaked & Genin, 2020; Shlesinger & Loya, 2016). The population of *Dipsastraea favus* exhibited positive population growth (i.e. λ > 1) and is predicted to gradually increase, whereas the population of *Platygyra lamellina* exhibited negative population growth (i.e. λ < 1) and is predicted to gradually decrease. It appears that *P. lamellina* is going through a population decline because of the lower rates of survival and recruitment than *D. favus*. The differences in recruitment are likely to be caused by the differences in spawning synchrony and fertilization success among these species (Shlesinger & Loya, 2019). For both populations, large colonies had a greater influence on population trajectories than small colonies.

As expected in corals with a massive growth form, the growth rates of both species were slow, and in the 3‐year study period (from 2015 to 2018) the increase in colony diameter averaged less than two centimetres. Nonetheless, perturbation analyses revealed that the population growth rates (λ) of both species were most sensitive to changes in colony growth, followed by changes in reproduction. As both the survival and reproductive potential of corals increase rapidly with colony size, individual colony growth effectively influences all other demographic processes. Together with the relatively low recruitment rates observed in this study, especially for *P. lamellina*, the finding that colony growth was most influential on the population growth rate was not unexpected. However, given that reduced coral growth and calcification might be associated with disturbances such as ocean warming and acidification (Cantin et al., 2010; Comeau et al., 2014; Pratchett et al., 2015), our results suggest that future alterations of coral growth rates may have substantial negative consequences on overall coral population dynamics.

The probability of coral colonies being fertile, the proportion of gravid polyps within the corals, and colony fecundity increased with colony size. Nonetheless, a variety of environmental and physiological stressors can reduce coral fecundity, gamete quality and fertilization success (Feldman et al., 2018; Hartmann et al., 2018; Liberman et al., 2021; Omori et al., 2001; Paxton et al., 2016; Ward et al., 2000). Reductions in fecundity or fertilization rate can further result in reductions in coral recruitment, leading to population decline. Similarly, as coral fertilization and subsequent recruitment are largely density dependent (Levitan et al., 2004; Nozawa et al., 2015; Oliver & Babcock, 1992; Teo & Todd, 2018), changes in adult abundances can also result in parallel changes in coral recruitment (Doropoulos et al., 2015; Gilmour et al., 2013; Hughes et al., 2019). While acute disturbances such as thermal stress can completely terminate or significantly reduce coral reproductive output for more than 2 years after a thermal stress event (Johnston et al., 2020; Levitan et al., 2014), chronic disturbances can also have deleterious effects on coral reproduction (Hartmann et al., 2018; Loya et al., 2004; Tomascik & Sander, 1987). In the present study, the estimates of coral reproductive parameters were based on data collected ca. 3 decades ago. Since fecundity may have declined through that 30‐year period, we simulated reductions in fecundity and examined the impact that such reductions may have on the rate of coral population growth. While reduced fecundity lowered the rates of population growth of both species, its effect on the population of *D. favus* was more pronounced than its effect on the population of *P. lamellina*. These results, in combination with the elasticity and perturbation analyses, show that reproduction and recruitment had more impact on the rates of population growth of *D. favus* than on the rates of *P. lamellina*, further emphasizing species‐specific differences.

Overall, the two coral species studied here share many similarities in their life‐history traits and demographic rates—they are both slow‐growing, highly fecund hermaphroditic broadcast‐spawning species with a massive growth form, belonging to the same family Merulinidae. Coral species possessing such traits are generally regarded as ‘stress‐tolerant’, resistant species (Darling et al., 2012; Klepac & Barshish, 2020; Loya et al., 2001). Given the challenges of identifying coral species, particularly in the field, and that life‐history traits frequently express ecological functionality (Madin et al., 2016; Mouillot et al., 2013), there is an increasing interest in heuristically categorizing corals for comparative purposes. For example, some studies emphasize ‘weedy’ versus ‘non‐weedy’ (Knowlton, 2001), or massive versus branching morphologies (Álvarez‐Noriega et al., 2016; Comeau et al., 2014; Klepac & Barshish, 2020; Loya et al., 2001; Pisapia et al., 2020), or some other multi‐dimensional trait measures (Darling et al., 2012; Denis et al., 2017; González‐Barrios et al., 2020; van Woesik, Franklin, et al., 2012). In most cases, studies using trait categories largely aim to estimate ‘winners’ and ‘losers’ (sensu Loya et al., 2001), or differential functionality following disturbances and ecosystem‐wide changes (Denis et al., 2017; González‐Barrios et al., 2020; Mouillot et al., 2013). While such approaches yield valuable insights, they amalgamate species (or even genera), which may result in the loss of subtle yet different species‐specific responses, as shown here. For example, branching corals of the highly speciose genus *Acropora* are generally recognized as highly susceptible to thermal‐stress (Graham et al., 2015; Loya et al., 2001; Mies et al., 2020), yet species level responses may vary considerably (Muir et al., 2017; van Woesik et al., 2011). Similarly, both species studied here would be classified as ‘stress‐tolerant’ or resistant coral species. Nonetheless, the two species displayed contrasting trajectories—one population was thriving whereas the second population was declining. Thus, although grouping corals by genera, functional traits or morphology can be appealing and productive from several aspects, it also masks subtle species‐specific trends that can have major ecological repercussions.

In recent years, many coral reefs throughout the world have suffered from mass bleaching events, however, the reefs in the Gulf of Eilat and Aqaba, in the northern part of the Red Sea have not shown such responses—in large part because of the unusually high thermal tolerance of the corals in this region (Bellworthy & Fine, 2017; Fine et al., 2013; Osman et al., 2018). As such, this region is regarded as a potential climate‐change refugium. Moreover, some of these northern Red Sea reefs even recovered following decades of anthropogenic stressors because of effective management that reduced many of the past stressors (Shlesinger & Loya, 2016). Nonetheless, ongoing increases in anthropogenic pressures together with the changing climate are threatening this coral‐reef ecosystem (Genin et al., 2020; Kleinhaus et al., 2020; Reverter et al., 2020; Rosenberg et al., 2019; Shlesinger & Loya, 2019) and may cause subtle, slow‐acting chronic changes that can eventually lead to local or regional declines in coral populations. Given the challenges of repeated sampling of the exact same areas, populations and colonies on the reef, our study represents a relatively restricted spatial extent. Therefore, it might be possible that the populations of the studied species will show different trends in other habitats or sites. Similarly, although this study spanned a 3‐year period in which the overall annual coral community recruitment rates were similar (Shlesinger & Loya, 2019), it is plausible that on a longer timeframe, recruitment rates will vary, and there might be years with sporadic high recruitment events that could counter the population decline.

Moreover, it is important to note that the projections of our models are only as good as the conditions under which they were constructed. Therefore, if the environmental conditions on the reefs deteriorate in the future, then so will the population growth rates. Cant et al. (2020) found that the population growth rates of some coral groups were substantially reduced during an acute thermal‐stress event, or under simulations of possible future ocean conditions. For example, they found that *Pocillopora* had a population growth rate value (λ) of 0.812, which declined to 0.299 the year following a thermal‐stress event. By contrast, *Turbinaria* showed little change over time (Cant et al., 2020). These findings, together with our results, emphasize the need to augment ecological studies and status assessments with species‐specific demographic approaches to fully understand the changes that are occurring on coral reefs.

With the developments of image‐based techniques assembling photomosaics and 3D reconstructions (Pedersen et al., 2019; Rossi et al., 2020; Yuval et al., 2021), the ease of monitoring specific coral colonies and reef plots along large spatial and temporal scales is growing rapidly. Accordingly, long‐term studies can benefit tremendously from incorporating these techniques to capture and derive valuable demographic information (Edmunds & Riegl, 2020). Yet, the identification of corals at the species level can be a challenging task that would restrict the feasibility of collecting species‐specific demographic data. Therefore, it might be useful to focus on a limited set of key species that could be relatively easy to identify. Indeed, detailed demographic information at the species level can provide early warning signs of population declines. In turn, demographic approaches can be extended to provide a useful predictive tool for guiding management actions that are aimed to reduce anthropogenic pressures and maintain ecosystem biodiversity so that coral reefs can be preserved in perpetuity.

## AUTHORS' CONTRIBUTIONS

Both authors conceived and designed the study. T.S. collected data in the field and led the analyses and writing of the manuscript; R.v.W. contributed critically to analyses and revisions of the manuscript.

## Supporting information

Supplementary Material

## Data Availability

All data and code used in this study are available on Zenodo http://doi.org/10.5281/zenodo.4553503 (Shlesinger, 2021).
