# Supplementary material for: Different population trajectories of two reef‐building corals with similar life‐history traits
Source: J Anim Ecol. 2021 Mar 17;90(5):1379–89. doi: 10.1111/1365-2656.13463 (PMC8252767; doi:10.1111/1365-2656.13463)
Supplement: Supplementary file 1 — Supplementary Material [file JANE-90-1379-s001.docx]

Supplementary material for:

**Different population trajectories of two reef-building corals with similar life-history traits**

**T. Shlesinger**^1,*^ **and R. van Woesik**^1^

*Journal of Animal Ecology* (2021)

^1^Institute for Global Ecology, Florida Institute of Technology, 150 West University Blvd., Melbourne, Florida, 32901 USA

*Correspondence: Tom Shlesinger, tomshlez@gmail.com

**
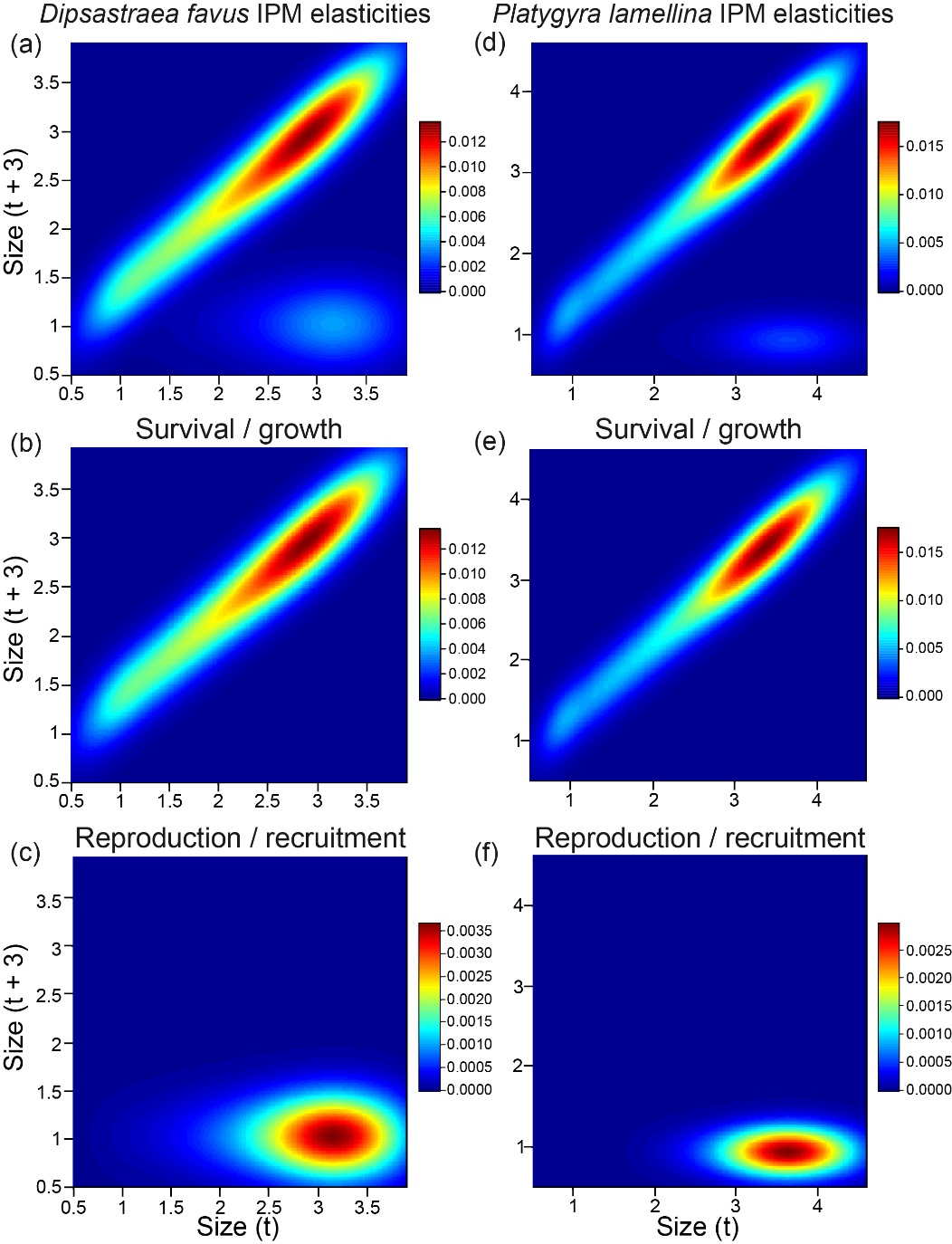
**

**Figure S1.** Elasticities of *Dipsastraea favus* (a – c) and *Platygyra lamellina* (d – f) on the reefs of the Gulf of Eilat and Aqaba, Red Sea from 2015 – 2018, split into contributions made by the survival/growth sub-kernel vs. the reproduction/recruitment sub-kernel. (a) and (d) depict the whole integral projection models’ elasticities. (b) and (e) depict the relative contribution of the survival and growth sub-kernels to integral projection models’ elasticities, and (c) and (f) are the relative contribution of the reproduction and recruitment sub-kernels.

**Table S1.** Reproductive data from Shlesinger (1985) for the two studied coral species, where n indicates sample size and SD indicates standard deviation.

| **Species** | **Size range (cm)** | **n** | **Diameter (mean)** | **Diameter (SD)** | **Percentage of reproductive colonies** | **Percentage of reproductive polyps (mean)** | **Percentage of reproductive polyps (SD)** |
| --- | --- | --- | --- | --- | --- | --- | --- |
| ***Platygyra lamellina*** | 1.01 – 5.00 | 1 | 3 |  | 0 | 0 |  |
|  | 5.01 – 9.00 | 10 | 7.85 | 1.05 | 80 | 14.32 | 9.59 |
|  | 9.01 – 13.00 | 10 | 10.6 | 1.15 | 90 | 32.93 | 18.14 |
|  | 13.01 – 17.00 | 10 | 15.41 | 1.16 | 100 | 46.84 | 23.27 |
|  | 17.01 – 21.00 | 10 | 18.16 | 2.28 | 100 | 70.21 | 29.72 |
|  | 21.01 – 25.00 | 10 | 22.33 | 1.06 | 100 | 93.75 | 9.82 |
|  | 25.01 – 30.00 | 10 | 29.05 | 2.56 | 100 | 96.52 | 16.85 |
| ***Dipsastraea favus*** | 1.01 – 5.00 | 8 | 2.91 | 0.63 | 37.5 | 18.75 | 9.38 |
|  | 5.01 – 9.00 | 10 | 6.18 | 0.85 | 70 | 55.76 | 15.1 |
|  | 9.01 – 13.00 | 8 | 10.69 | 1.48 | 75 | 63.33 | 25.82 |
|  | 13.01 – 17.00 | 8 | 14.28 | 0.52 | 87.5 | 68.39 | 20.24 |
|  | 17.01 – 21.00 | 8 | 18.53 | 0.92 | 100 | 75.02 | 11.07 |
|  | 21.01 – 25.00 | 10 | 24.32 | 1.33 | 100 | 93.2 | 8.8 |

**Table S2.** Parameter estimates used in integral projection models of *Dipsastraea favus* on the reefs of the Gulf of Eilat and Aqaba, Red Sea from 2015 – 2018. Since the survival regression intercept was not significantly different than zero, we forced the regression to go through the origin (i.e., intercept at zero). Where n indicates sample size, SE indicates standard error, and SD indicates standard deviation.

| **Model** | **n** | **intercept** | **SE** | **p-value** | **slope** | **SE** | **p-value** |
| --- | --- | --- | --- | --- | --- | --- | --- |
| Growth | 154 | 0.497 | 0.051 | 0.000 | 0.836 | 0.026 | 0.000 |
| Survival | 167 | 0.000 |  |  | 1.442 | 0.181 | 0.000 |
| Colony reproductive probability | 52 | -2.896 | 1.260 | 0.021 | 2.006 | 0.628 | 0.001 |
| Proportion of gravid polyps | 52 | -2.917 | 1.157 | 0.011 | 1.481 | 0.490 | 0.002 |
| Colony fecundity | 52 | 5.308 | 0.169 | 0.000 | 1.985 | 0.069 | 0.000 |
| Recruits | 35 | Mean size = 0.978, SD = 0.212 | | | | | |

**Table S3.** Parameter estimates used in integral projection models of *Platygyra lamellina* on the reefs of the Gulf of Eilat and Aqaba, Red Sea from 2015 – 2018. Since the survival regression intercept was not significantly different than zero, we forced the regression to go through the origin (i.e., intercept at zero). Where n indicates sample size, SE indicates standard error, and SD indicates standard deviation.

| **Model** | **n** | **intercept** | **SE** | **p-value** | **slope** | **SE** | **p-value** |
| --- | --- | --- | --- | --- | --- | --- | --- |
| Growth | 70 | 0.387 | 0.079 | 0.000 | 0.883 | 0.034 | 0.000 |
| Survival | 83 | 0.000 |  |  | 0.861 | 0.156 | 0.000 |
| Colony reproductive probability | 61 | -14.531 | 7.445 | 0.051 | 7.831 | 3.681 | 0.033 |
| Proportion of gravid polyps | 61 | -8.996 | 2.438 | 0.000 | 3.378 | 0.887 | 0.000 |
| Colony fecundity | 61 | 7.167 | 0.142 | 0.000 | 1.962 | 0.051 | 0.000 |
| Recruits | 8 | Mean size = 0.903, SD = 0.150 | | | | | |
